# The measuring aerosol spreading during countermeasures (MASC) study presents an automated system to investigate face mask efficacy and other aerosol countermeasures in varying environments

**DOI:** 10.1038/s41598-022-25210-5

**Published:** 2022-12-09

**Authors:** Johannes Schreiber, Dörthe Brüggmann, Markus Braun, David A. Groneberg

**Affiliations:** Institute of Occupational Medicine, Social Medicine, Environmental Medicine, Goethe University Frankfurt, Theodor-Stern-Kai 7, 60590 Frankfurt Am Main, Germany

**Keywords:** Environmental sciences, Public health

## Abstract

The COVID-19 pandemic exemplified the importance of research on personal protective equipment. In specific, understanding how effective surgical masks or particulate filter respirators are at reducing the transmission of infectious diseases has suddenly become one of the most pressing issues for legislators, regulators, and everyday life. However, there was a lack of available scientific platforms to assess this issue. Therefore, we designed and built a system entitled MASC: measuring aerosol spreading during countermeasures. This platform allows the simulation of various everyday situations and evaluation of the efficacy of masks and respirators in reducing the amount of inhaled particulate matter from the air. Furthermore, MASC can be used to investigate how aerosols propagate in closed spaces, such as offices or classrooms. It can be used to generate aerosols on command and control the room temperature, humidity, and wind speed. Up to four laser aerosol spectrometers can be read simultaneously, and a camera can automatically take pictures to evaluate the efficacy of countermeasures to prevent the spread of aerosols. The aerosol generation, measurement periods, and the number of repetitions for an experiment can be configured digitally and are executed by a computer automatically. A website displays the data in real time and allows monitoring of the experiment. Upon completion, statistical values are calculated automatically to accelerate the evaluation of the gathered data. Codes and technical drawings in this present methodology publication are open source and can be used by the scientific community to establish similar systems.

## Introduction

With the beginning of the COVID-19 pandemic, the worldwide interest in the efficacy of personal protective equipment (PPE) and specifically masks and respirators as protection against airborne diseases has suddenly skyrocketed. A search on Clarivates Web of Science with the term “(mask OR respirator) (efficacy OR efficiency)” shows 1399 and 1776 results published in the years 2020 and 2021, respectively, while previous years did not reach more than 1000 publications. However, computer automation to collect large amounts of reliable data seems to be rarely used in published articles. Furthermore, many experiments were set up in fairly small, dedicated measuring chambers^1–8^, or just tested the mask material in isolation^9–11^. Thus, these approaches may not accurately reproduce effects such as air turbulence, which would naturally occur in situations where people wear masks. Up until recently, there were only a few experiments that dealt with mask efficacy or the spread of aerosols in realistic scenarios, such as concert halls^12^, small rooms^13–15^, vehicles^16,17^, or classrooms^18–20^. These experiments presumably took a lot of time to set up or operate. Some of the more time-consuming approaches to collect data include using agar cultures^21^, measuring fluorescein levels^22^, or taking photos of droplets on water-sensitive paper^23^. Using human volunteers makes it even more costly to get large amounts of reliable data (typically, only a handful of subjects are measured for a few minutes^24–26^), and different face shapes make comparing results from various studies difficult. While human studies might arguably be ‘more realistic’, they also make it difficult to rapidly change experimental parameters such as mask tightness in a very controlled way and quantify their effects.

We aimed to design and establish a platform to perform high throughput testing of mask efficacies under varying environmental conditions. In contrast to existing studies, our approach should not only be usable in realistic settings, such as classrooms, offices, or laboratories but also give very consistent, repeatable, and accurate results. After an initial setup time, which typically takes us less than half an hour, an experiment can be repeated as many times as desired without human interaction. To comply with ethical guidelines and to facilitate the operation of the platform, we decided to abstain from the inclusion of animal or human testing.

## Methods

### Creating and removing aerosols

The MASC platform is not restricted to definite technologies to create aerosols. For precision and repeatability, a Portable Test Aerosol Generator TSI model 3073 with Diethylhexyl sebacate (DEHS) is used to generate a stable polydisperse submicron aerosol. The size distribution extends to around 2.5 µm, above which almost no aerosol is generated. The aerosol generator is connected to 1 m of plastic tube, which exits a manikin head through the mouth.

A TTV 4500 Industrial Floor Fan by Trotec is integrated into the system and can be used to create a more consistent stream of air, which counteracts the convection of air in the room and creates very repeatable results. This approach alters the natural flow of the aerosol, so the fan will only be used in certain experiments. To produce the same conditions before every experiment, we use a TAC V+ high-performance air purifier by Trotec with an H14 HEPA filter, capable of filtering 1200 m^3^ of air per hour. Starting each experiment in a particle-cleaned room also ensures that our measurements are not tainted by varying environmental aerosol concentrations.

### Measuring aerosol concentrations, particle sizes, and mask efficacy

**Figure 1:**
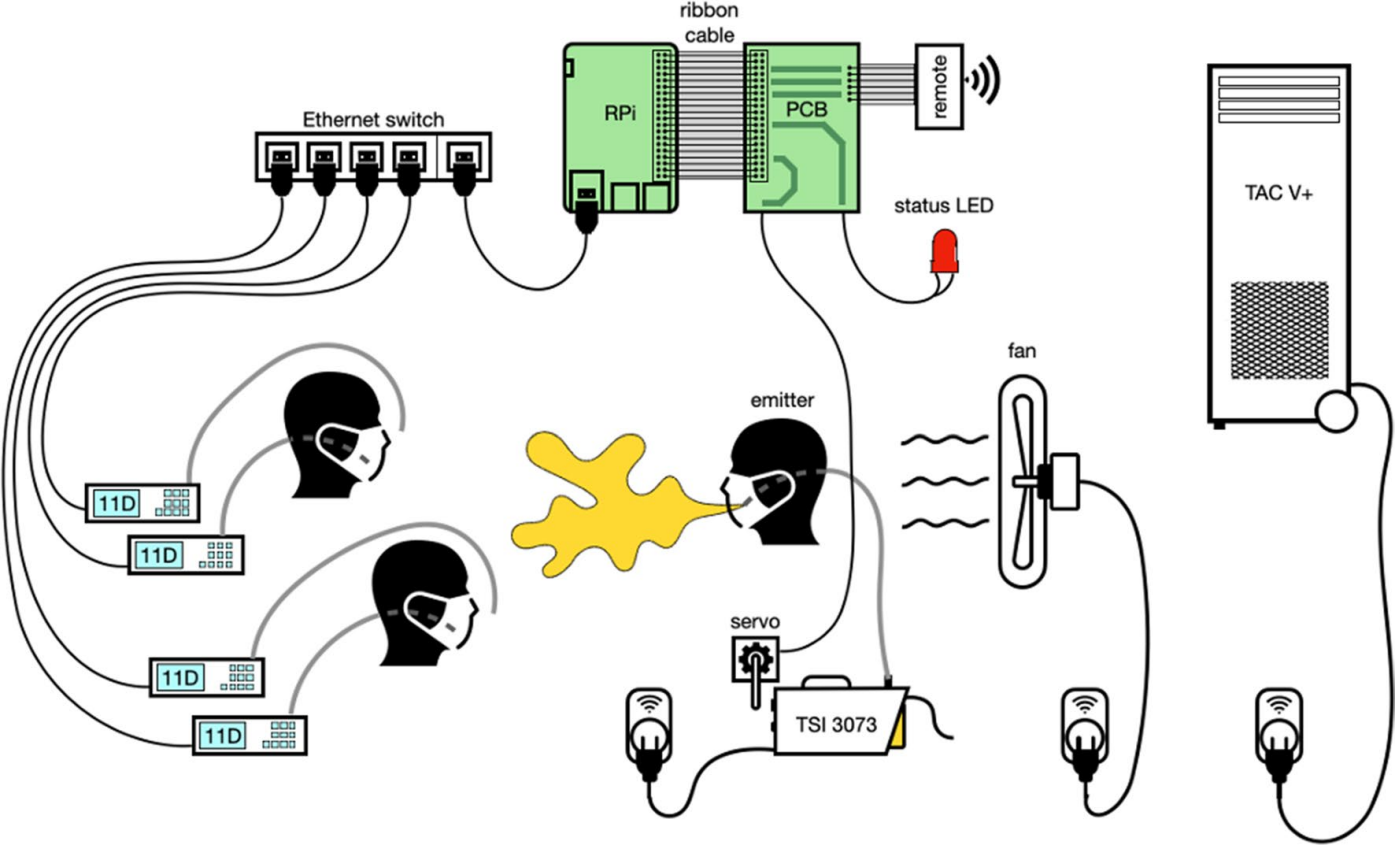
Schematic figure of a possible experimental setup. The figure shows four connected aerosol spectrometers (LAS), the controlling single-board computer (SBC), the custom printed circuit board (PCB), the aerosol generator, the air purifier, and a fan to create a uniform stream of aerosols.

Four simultaneously operating Grimm 11-D laser aerosol spectrometers (LASs) are used to measure and quantify aerosol concentrations. They can detect particles between 0.25 and 35 µm in size and particulate matter (PM) concentrations up to 10^5^ µg/m^3^. Measurements are made every 6 s with an inflow of 1.2 L per minute. Each LAS is connected to one meter of flexible plastic tube, which—depending on the experimental setup—can be located at the nose or mouth part of a dummy or be left bare, as shown in Fig. 1. PM_2.5_ and the total number of particles are used to evaluate the efficacy of a given mask because the aerosol generator barely produces particle sizes over 2.5 µm, so the PM_10_ values are essentially the same as PM_2.5_.

Currently, the terminology “mask efficacy” is used to denote the percentage of aerosol that is removed by the mask during inhalation. Let *c*_*o*_ be the concentration of aerosol outside of the mask, and *c*_*i*_ be the concentration of aerosol inside (behind) the mask, then the mask efficacy *e* is given as

1 $$e = \left(1-\frac{{c}_{i}}{{c}_{o}}\right)*100\mathrm{\%}$$

### Automation

**Figure 2:**
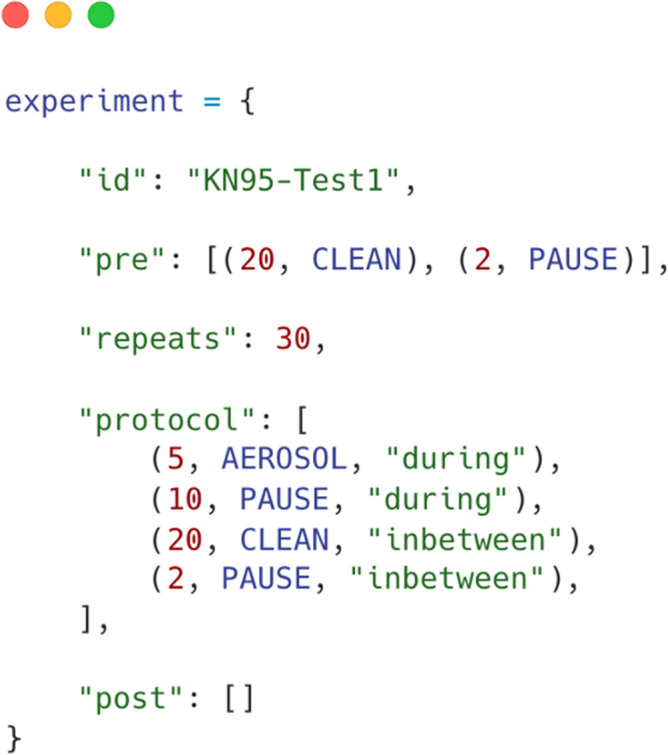
Configuration file for experiments. Explanation of Python file elements: “id” identifies the data uniquely. “pre” are actions run once before the experiment starts. “repeats” is how many times the experiment runs. “protocol” contains the sequential steps that are performed for the experiment. “post” contains actions that are performed once after the experiment finishes. The notation (20, AEROSOL, “during”) specifies that aerosol should be generated for 20 min. “during” is a comment that is added to the CSV data file. Similarly, CLEAN turns the air purifier on, and PAUSE does nothing except continue to collect data. Measurements are made every 6 s automatically and don’t have to be configured.

**Figure 3:**
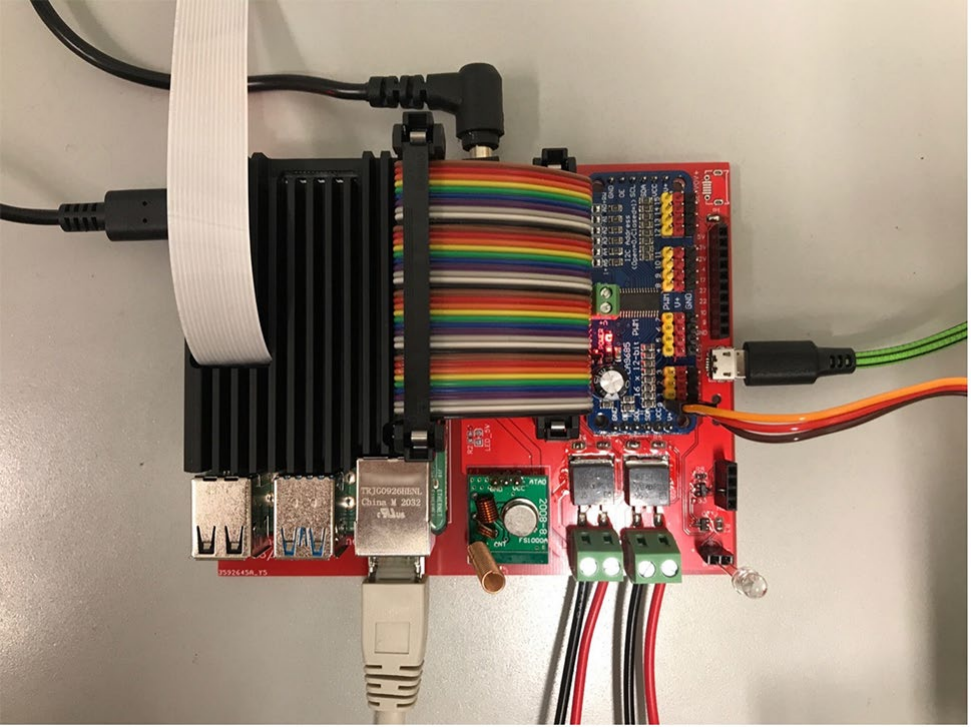
The hardware for controlling the experiments. The red printed circuit board (PCB) is the one designed by the authors, connecting the single-board computer (SBC) with everything else. The blue PCB is a servo controller, which drives the servo motor to turn on the aerosol generator. The green PCB is a 433 MHz transmitter, which can switch power outlets on or off, which are connected to the air purifier, for example.

To automate the experiments as much as possible, a Raspberry Pi 4 Model B single-board computer (SBC) is used, powered by over 1200 lines of Python code that is publicly available. For each experiment, a sequence of steps is defined in a separate python file that describes what actions to perform and how many times they should be repeated, as shown in Fig. 2. All LASs are connected via ethernet cables to a network switch, which is in turn connected to the SBC. The Modbus TCP protocol is used to retrieve data from each LAS, and the data is logged to a comma-separated values file (CSV) every 6 s precisely. The air purifier, aerosol generator, and fan are turned on and off via 433 MHz radio-controlled sockets. The aerosol generator required a servo motor controlled by the SBC via pulse-width modulation (PWM) to turn on the device. Figure 3 shows the custom printed circuit board (PCB) we designed to hold the SBC, a 433 MHz transmitter module, infrared LED, and 12-Volt screw terminals, which can be switched on/off. These can be programmed to control a wide range of commonly used devices such as air conditioning or solenoid valves to change the environmental conditions and redirect the flow of aerosols. The screw terminals can also be used to power a laser to illuminate the produced aerosol.

A connected webcam can take photos every 0.5 s or continuous video footage to document the experiment and the flow of aerosols. A still image is taken beforehand and automatically subtracted from every image, therefore removing nearly everything that is not aerosol. The Canny edge detection algorithm is applied automatically and overlayed on the final video, which makes it easier to see the experimental setup without adding too much distraction. After an experiment is finished, the software automatically calculates statistical values, such as the mean and standard deviation of the number of particles and PM_2.5_ values, which can be imported afterward into third-party statistical software for further evaluation.

**Figure 4:**
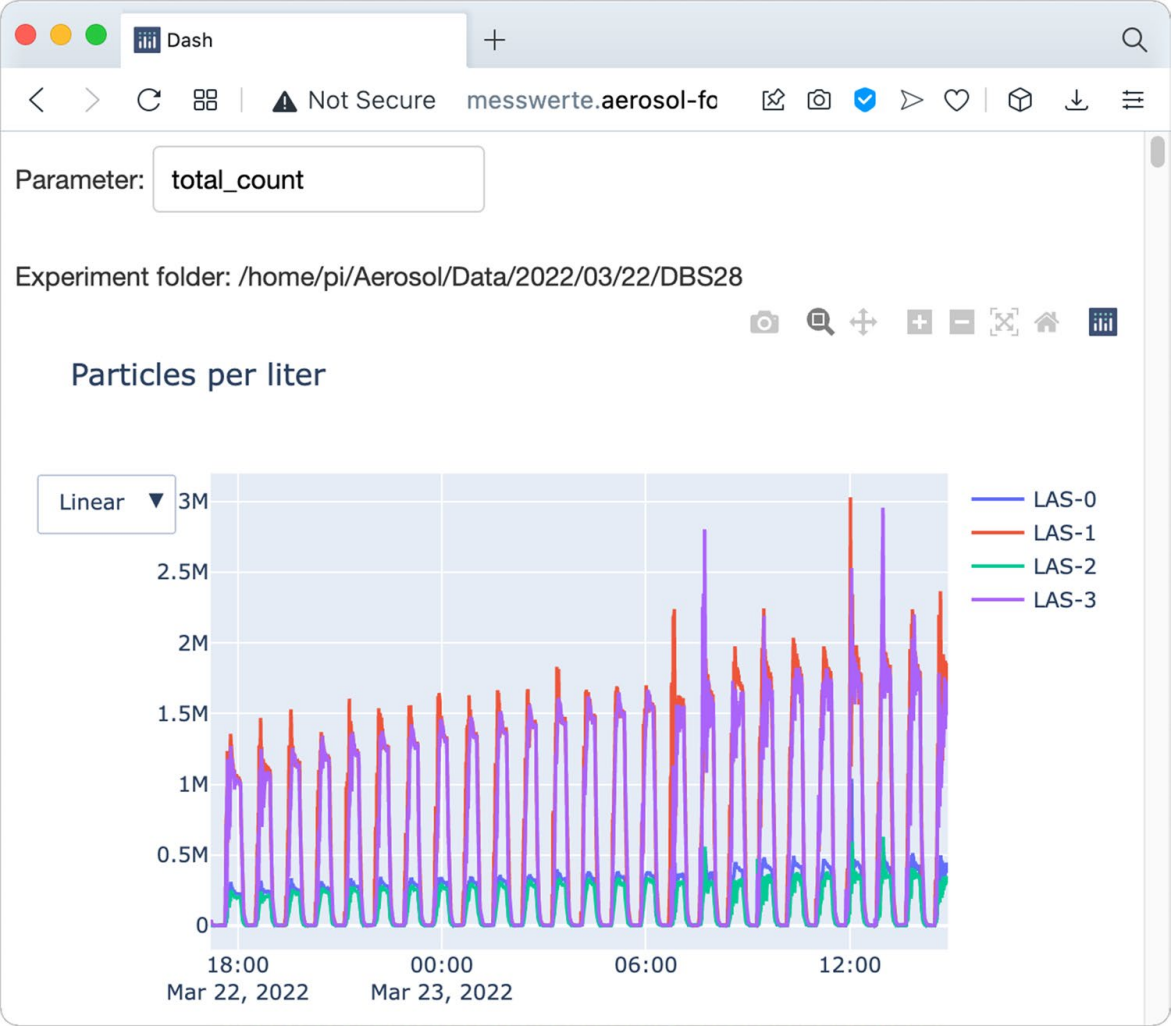
A website to supervise experiments. We created a website (here: exemplary image) running locally on the single-board computer to show the incoming data in real time. That can be used to quickly check the effect of a certain countermeasure and ensure the experiment’s success.

Furthermore, we created a website using the “Plotly Dash” framework for Python to remotely monitor the readings from all devices (Fig. 4).

### Variations in mask position and fit

**Figure 5:**
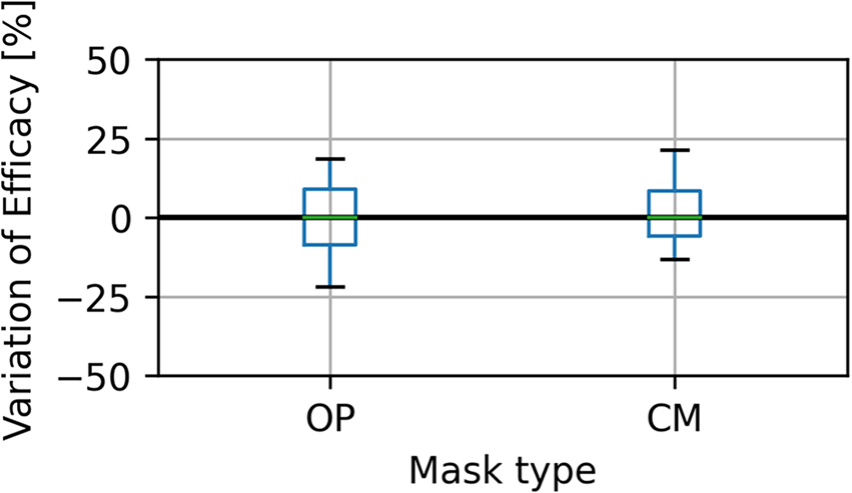
Variations between several masks from a single charge for surgical (OP) and community (CM) masks. The boxes show the Q1 to Q3 quartiles of the data, and the whiskers the maximum values. The variation is given in percentage points added to or subtracted from the mean efficacy of that mask type, so the doffing and donning of a fresh mask usually leads to a ± 10% absolute difference in the observed mask efficacy.

Reliably placing the mask the same way each time is necessary to create repeatable results. However, sealing the mask to the face is not a good “real world” model where getting a perfect fit is very difficult. So, we decided not to tape the masks to the dummy heads or otherwise create a perfect seal, instead doing more realistic measurements which include imperfections in the mask fit and thus are closer to the actual efficacy in real usage. To quantify the variations in fit, we applied a mask to the manikin, measured its efficacy for one minute, and then took it off again. That was repeated 20 times for each mask type. The resulting distribution is shown in Fig. 5, which demonstrates a difference of roughly ± 10% in the mask efficacy due to the impossibility of donning a mask exactly the same way every time.

### Mask efficacy as a function of concentration

**Figure 6:**
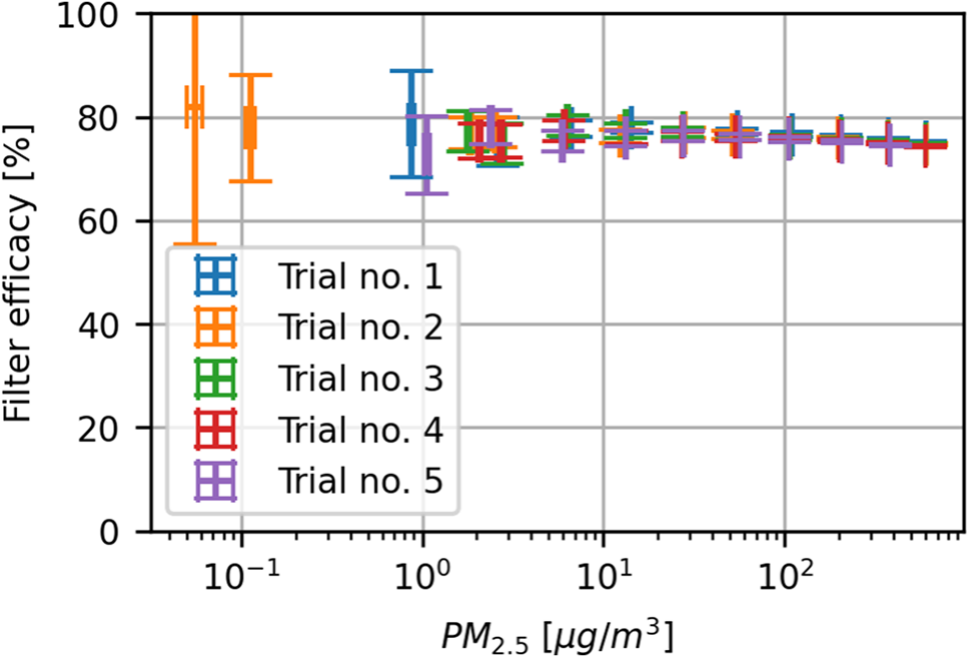
Efficacy of a single FFP2 mask as a function of surrounding aerosol concentration. Five measurements (trials 1 to 5) were made. One trial consists of multiple measurements at increasing concentrations. The concentrations were increased by simply generating aerosols for a longer period. The brackets show the 95% confidence intervals of 100 data points. Although there seems to be a slight reduction in efficacy at higher concentrations, the overall effect is very small, and the efficacy is nearly constant at all concentrations. Very similar trends were observed with surgical masks (not shown).

To get a better signal-to-noise ratio between the presently generated aerosols and the residual particles in the room, higher concentrations are used than aerosols exhaled by humans. According to Holmgren et al.^27^, who used a similar LAS from Grimm, up to around 10,000 particles are exhaled per liter of air, while we are often exceeding 1,000,000 particles per liter. We had to show that the filtration efficacy of masks does not change within reasonable limits when the outside aerosol concentration varies. Figure 6 illustrates how the measured mask efficacy changes as a function of aerosol concentration. The variation is small enough, that for the present purposes we assume that our measurements at high concentrations are also representative of mask efficacy at lower concentrations.

### Room sizes and setup

For the establishment of the MASC platform, experiments are mainly conducted in a laboratory room that measures 3 × 3 × 3 m. Of course, this system is not restricted to a definite room or setup and can be transported to whatever location. However, we noticed that it is advantageous to remove spaces where aerosols or particles can remain or be trapped, e.g., taping over spaces behind cupboards, because this reduces the amount of time it takes the air purifier to clean the room, and it reduces residual contamination. Also, masking tape should be placed over all sinks and window cracks, reducing pollution through outside air, and rubber gaskets should be installed around the door, minimizing air exchange.

### Fan usage, wind speeds, and environmental parameters

**Figure 7:**
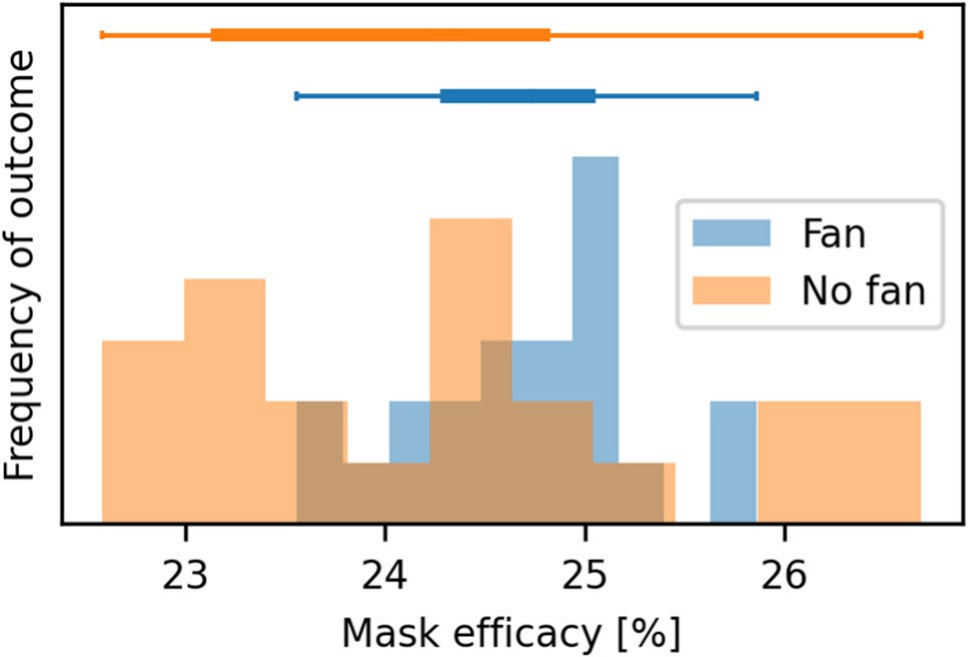
Differences in measurements when using a fan behind the emitter. The plot shows 22 repeated measurements of surgical masks' efficacy on the receivers' head. The blue values were recorded with a fan behind the emitter. The horizontal boxplots on top show the distribution of the first and third quartile (solid box), while the whiskers show the minimum and maximum values. The Wilcoxon signed-rank test and Kruskal–Wallis-Test showed no significant difference (*p* > 0.11). That means that using a fan behind the emitter to gently blow the aerosol towards the receiver does not significantly alter the measured efficacy.

To standardize air currents and create more uniform results, a fan behind the aerosol emitter can be used. That generates wind speeds of 2.8–3 m/s right in front of the fan, 0.6–1 m/s at a distance of 1 m, and 0.4–0.6 m/s at 2.2 m. Certainly, that strongly impacts how aerosols distribute in an enclosed space. However, it does not significantly change the measurement of the receiver’s mask efficacy, as Fig. 7 shows. Placing the fan anywhere else in the room can be used to generate turbulences, simulating rooms with more air movements. If the 1158-TRH sensor by Grimm is connected, temperature, humidity, and barometric pressure values are automatically recorded, too. Temperature and humidity in the room can conceivably be adjusted by connecting, e.g., an air conditioning unit, electric heater, or dehumidifier using 433 MHz remote-controlled sockets. This approach is already used to control the aerosol generator, fan, and air purifier, so the changes to the source code would be minimal. Because we use DEHS (which is hydrophobic and evaporates extremely slowly) in the evaluation of the system, we have not yet employed this strategy.

### Reliability and device calibration

Because the LASs don’t measure absolutely identical values, calibration factors are applied, which are determined before every change in setup or aerosol concentration. For a sufficiently large n, let m_1_ be the median of n measurements of LAS 1 and m_2_ be the median of LAS 2. Then the calibration factor for LAS 1 is calculated as m_2_/m_1_. When multiplied with the measurements of LAS 1, then LAS 1 and LAS 2 will produce the same results when measuring next to each other and can be compared to one another. The number n depends on the setting and desired accuracy. In our setup, n = 450 typically resulted in calibration factors accurate to within around 1% (95% confidence interval), so, for example, if LAS 1 measured 6% fewer particles than LAS 2 (this is the maximum error we observed with our LASs), the correction added to LAS 1 would be + 6%, although the *real* correction, which we may never know exactly, must be between + 5% and + 7% (with 95% probability). Calibration factors are always applied to the LASs, which are not behind a mask. However, we want to point out that even without correction, the influence on the results is very small because the 6% error is not *added* to the measured mask efficacy but rather multiplied by the measurements in front of the mask. That means that the absolute introduced error tends to zero, as the mask efficacy tends to 100%.

During an experiment, we measure one setup at least 20 times and calculate the mean concentration for each repetition. That typically leads to normally distributed data that is used for subsequent further analysis.

### Aerosol size distribution

The particle sizes of the aerosol are measured using the four aforementioned LASs. The peak of the size distribution is at about 250 nm and was verified by a TSI Scanning Mobility Particle Sizer. The largest particle diameter we chose to include in our evaluation is 2.53 µm because the aerosol generator barely produces any DEHS aerosols beyond that size, which is also why we mainly use PM_2.5_ values instead of PM_10_. That covers the particle range typically produced by human breathing according to Bake et al.^28^.

## Results

Here we show some exemplary results and how the MASC system can be used.

### Removing aerosols from the room

**Figure 8:**
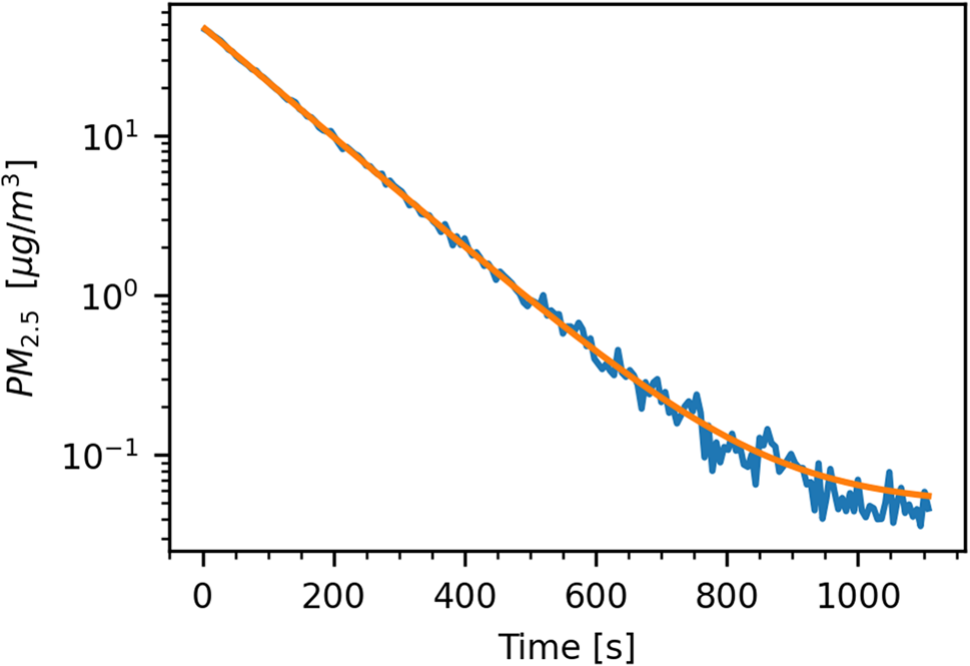
Removal of aerosols from the room. The original data is shown in blue. The orange curve is an exponential model fitted to that data.

**Figure 9:**
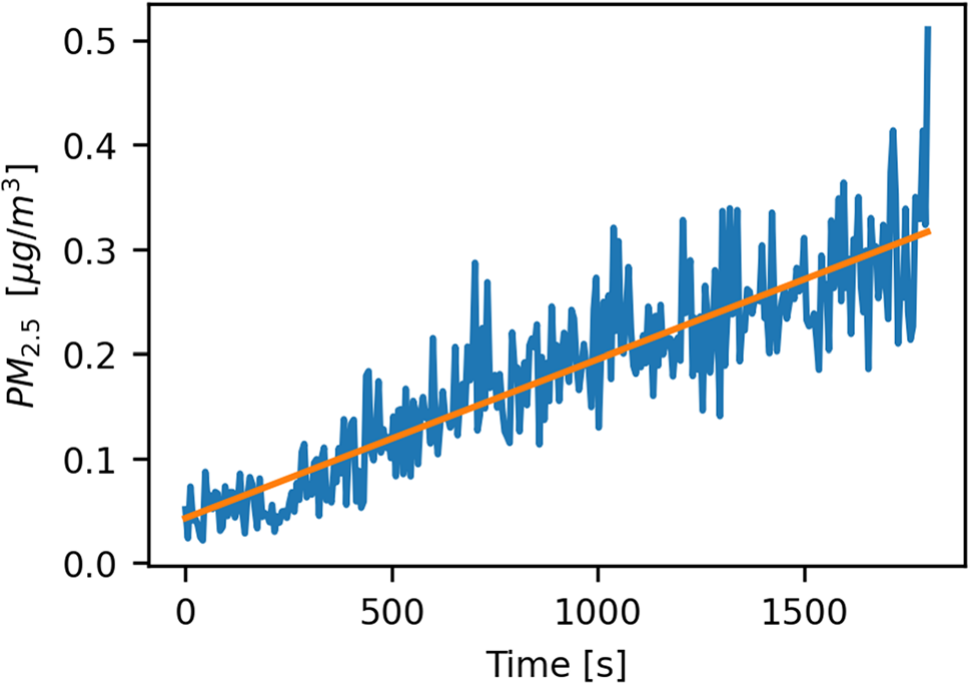
Rise of the aerosol concentration within 30 minutes after cleaning the room. The data was collected without any other interference. The measured concentration is shown in blue, and the fitted linear model is in orange.

We determined the efficacy of the air purifier in our laboratory room and fitted the resulting data with an exponential function of the form

2 $$C\left(t\right) = a\times {10}^{-bt} + c$$

where *C* is the PM_2.5_ concentration in µg/m^3^, *t* is the elapsed time in seconds, *a* + *c* is the starting concentration at the beginning of the experiment (*t* = 0), *1/b* is the time in seconds it takes to reduce the aerosol concentration to one-tenth of its original value, and *c* is the lowest possible concentration we can reasonably achieve as *t* gets very big. The resulting function describing the aerosol concentration *C* as a function of time *t* is

3 $$C\left(t\right)=100\times {10}^{-0.0035t}+0.04$$

showing that it takes the air purifier about 285 (= 1/0.0035) seconds to reduce the aerosol concentration by 90% and 20 minutes to reach a concentration of 0.046 µg/m^3^ PM_2.5_. Figure 8 shows the decline in aerosol concentration during this operation. When the air purifier is turned off, the concentration of particles rises again. For around 30 min, the increasing concentration can be modeled by a linear function

4 $$C\left(t\right)=0.04+0.00015t$$

where again *C* is PM_2.5_ in µg/m^3^ and *t* is the time in seconds since the air purifier turned off. These concentrations are usually less than 1% of the generated aerosol concentrations in our experiments, influence all devices by the same amount, and get reduced by masks the same amount, so we decided not to account for this effect. Figure 9 shows the rising aerosol concentration after the air purifier is turned off.

### Automatically captured images

**Figure 10:**
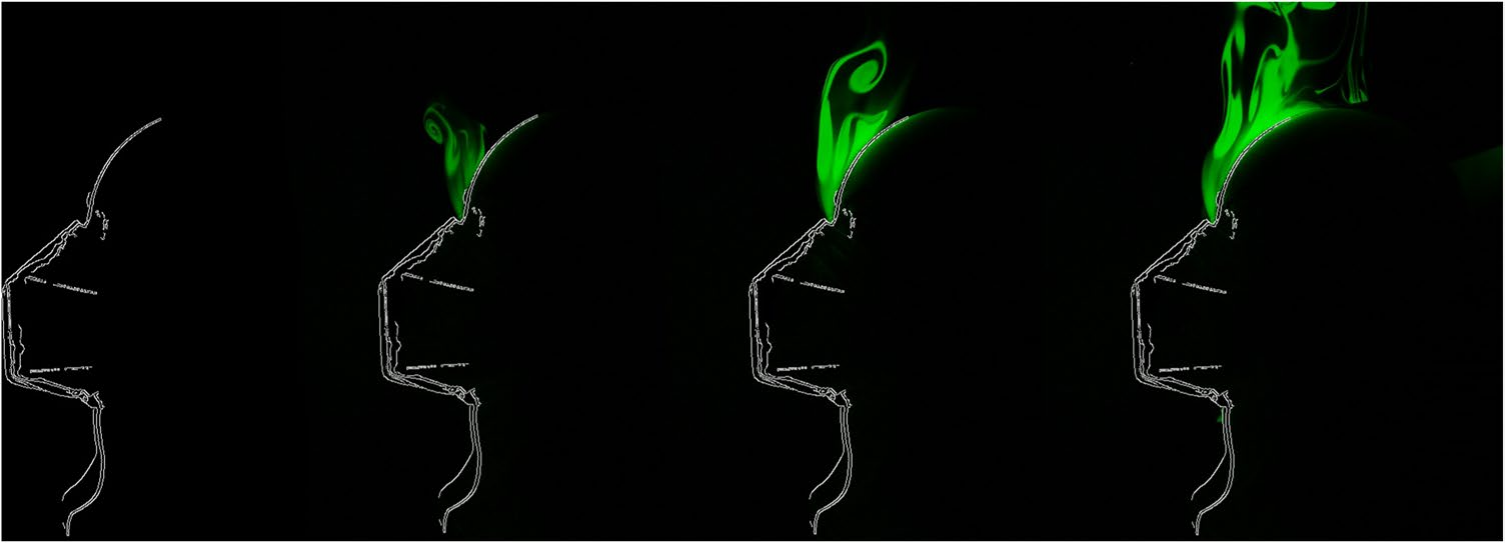
A sequence of four images taken automatically during an experiment a few seconds apart. DEHS aerosol is expelled from behind the mask. Illumination was provided by a 50 mW line laser module (wavelength 530 nm). All images had a still image subtracted from them to show only the aerosol and not the background. Additionally, a Canny edge detection filter was applied to better show the geometry of the head.

Figure 10 shows an example of images the system can capture. After pointing a green 50 mW line laser at the manikin and setting up the webcam, the SBC automatically took images and did the post-processing to enhance the visibility of the aerosol. The image shows that in the medial plane, the maximum leakage of an FFP2 mask occurs around the glabella rather than at the chin or through the mask.

### Example data from one experiment

**Figure 11:**
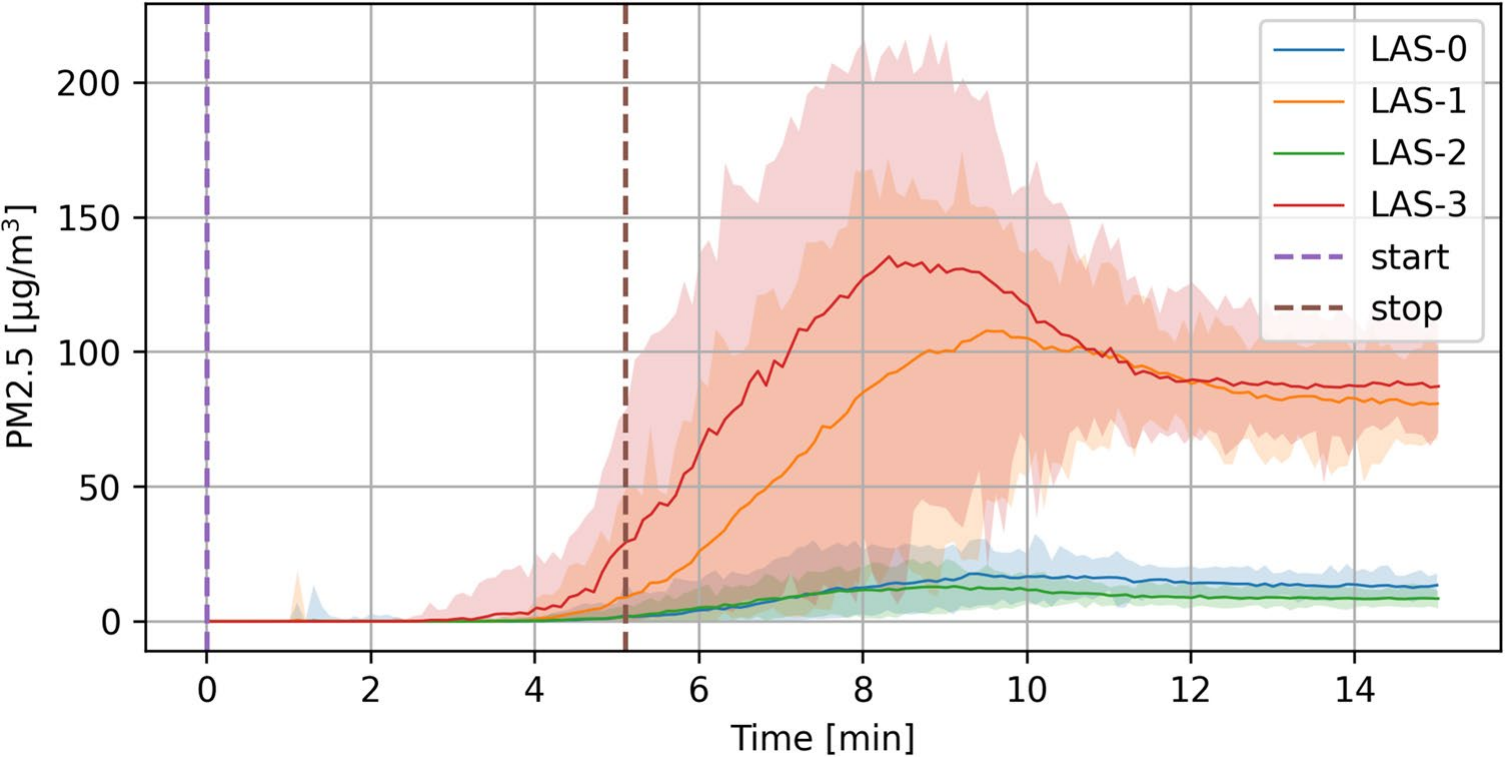
Example data of 40 repeated experiments. Solid lines show the mean concentration at each point in time. The shaded areas indicate where 95% of the actual measurements lie. Aerosol emission starts at the purple dashed line and stops at the brown dashed line. LAS-0 and -1 were at a 1 m distance, and LAS-2 and -3 at 2.5 m. LAS-0 and -2 had tightly fitting FFP2 masks on, LAS-1 a loosely fitting FFP2, and LAS-3 no mask at all.

Figure 11 shows a typical result when gently blowing aerosol into our 3 × 3 × 3 m laboratory room with no forced air movement. LAS-0 was placed at a distance of 1 m from the emitter with a tight-fitting FFP2 mask, LAS-1 at 1 m with a loose-fitting one, LAS-2 at 2.5 m with a tight-fitting one, and LAS-3 at 2.5 m with no mask. It takes around three minutes until the concentration rises visibly at a distance of one meter and around four minutes at a distance of two meters. After stopping the aerosol generation, it takes another three to four minutes until the peak concentration is reached. The graphs for 20 repetitions show quite a bit of variance (indicated by the shaded areas), presumably because residual convection sometimes carries the aerosol straight towards the emitter and sometimes off to the side.

While wearing an FFP2 mask, increasing the distance from one to 2.5 m leads to a reduction of inhaled aerosol mass of 23.4% (18.2–28.6%, 95% confidence interval). At 2.5 m, wearing an FFP2 mask reduced aerosol intake in this experiment by 90.5% (90.2–90.7%).

### Efficacy of commonly used medical masks and respirators

We tested surgical masks, N95/KN95/FFP2, and FFP3 respirators from eight different manufacturers each. Each mask was fitted to the receiver’s head. The emitter was placed at a distance of 1 m. The reduction of PM_2.5_ was used to calculate the mask efficacies according to Eq. 1. The worst-performing surgical mask had an efficacy of 13.3%, the best-performing one reduced aerosol concentrations by 37.7%. For N95, KN95, and FFP2 respirators, the worst and best ones were measured at 16.9% and 70.3%, respectively. FFP3 respirator ranged from 45.8 to 95.9% efficacy. For reference, a publication testing the influence of fit on mask efficacy measured surgical masks at around 20% and KN95 at < 40% under similar circumstances^3^.

## Discussion

The benefit of the presently established MASC platform system is the possibility of investigating many different setups and measures used to reduce aerosol transmission with ease.

Of previously published studies similar to our one^12,13,15,17,18^, none seem to employ automation like we do, as they are “one-off” experiments.

A limitation of our current setup is that physiological breathing cannot be simulated yet. That may affect air currents and the distribution of aerosols. However, due to the easy expandability of our system, a breathing simulator can be added at a later stage easily. With easily connected solenoid valves between the aerosol generator and the emitter, the emission of aerosols can be quickly turned on and off to approximate human breathing patterns, but without inhalation.

The flow rate of 1.2 L/min of the used LASs is much lower than human breathing, which can reach a few liters per second. That creates less suction, so there is a chance we are underestimating the actual efficacy of tightly fitting masks, which could get pulled closer to the face at higher inspiratory rates, thus creating a better seal.

We observed that the mask fit is an incredibly important factor in how well a mask performs. Even the slightest gap greatly reduces fine particles' filtration efficacy, although there is likely still good protection from larger ballistic droplets. Importantly, it seemed that getting a good fit around the nose and glabella was the most difficult, while the chin and neck rarely posed a problem. Figure 10 clearly shows this effect on an FFP2 mask.

The air purifier in our setup was also used by investigations of Kähler et al.^29^ and was deemed very effective for preventing the transmission of aerosols in schools and offices. Its throughput proved enough to remove 99.9% of particles in our laboratory room within 20 minutes, so the influence of previous experimental runs on a setup is negligible for our purposes.

Lastly, this setup is more suitable for investigating fine particles because the applied aerosol generator only really produces particles of < 2.5 µm, and ballistic droplets would not make it through the winding 1 m plastic tube. That makes it more suitable for the study of airborne diseases than droplet transmission.

## Conclusions

We established a platform titled MASC that allows measuring the efficacy of masks under a variety of circumstances, the fit of those masks, the efficacy of other preventative measures such as glass barriers, and the propagation of aerosols in larger spaces such as classrooms and offices. Due to the automation and easy programmability, data can be acquired with arbitrary precision when averaging over enough repetitions. A local website allows monitoring experiments in real time. The option to take photos and automatically remove everything that is not laser-illuminated aerosol creates visually pleasing and easy to understand images. Taken together, this amounts to large time savings when investigating aerosol-related issues. All code and hardware schematics are open source and can be used by other researchers.

Our most notable result when evaluating MASC was the measurement of different masks and respirators commonly worn in hospital and community settings. We showed that under realistic circumstances and with a suboptimal fit, the filtration capability of masks is drastically reduced. In our setup, surgical masks filter out only between 13–38%, N95/FFP2 respirators 17–70%, and FFP3 respirators 49–96% of particles between 0.25 and 2.5 µm.

## Supplementary Information

Supplementary Video 1.

## Data Availability

The source code and schematics are available at https://github.com/s8859231/masc. Data used to generate the diagrams in this publication are available from the corresponding author upon reasonable request.

## Abbreviations

CSV: Comma-separated values
DEHS: Di(2-ethylhexyl) sebacate
LAS: Laser aerosol spectrometer
PCB: Printed circuit board
PM: Particulate matter
PM_2.5_: Particulate matter less than 2.5 µm in diameter
PM_10_: Particulate matter less than 10 µm in diameter
PWM: Pulse width modulation
SBC: Single-board computer
TCP: Transmission control protocol
